# Supplementary material for: DROID: discrete-time simulation for ring-oscillator-based Ising design
Source: Sci Rep. 2025 May 28;15:18643. doi: 10.1038/s41598-025-00037-y (PMC12119999; doi:10.1038/s41598-025-00037-y)
Supplement: Supplementary file 1 — Supplementary Information. [file 41598_2025_37_MOESM1_ESM.pdf]

Supplementary Materials: DROID: Discrete-Time Simulation for Ring-Oscillator-Based Ising Design

S1. Pseudocode for the event-driven simulation algorithm

The simulator algorithm is described by pseudocode in Algorithm 1. For better readability, the steps in the algorithm, listed in the main manuscript, are reproduced below.

Step 1: Initialize Initial events at the enable cells are scheduled to start the ROs. The queue, Q, and the map, Net2Event, are populated to reflect these events, and PendingTrigger is initialized to an empty map.

Step 2: Pop and process an event The earliest occurring event E is popped from Q. The event is passed to the PROCESS_EVENT function which generates new events that result from E. Consider an event that occurs at the v_{in}^{f} pin of a coupling cell and the map Net2Event contains another event that occurs at h_{in}^{f} of the same cell. Then, PROCESS_EVENT will operate on these two events to generate events on output pins v_{out}^{f} and h_{out}^{f}, of the coupling cell. We will illustrate the functionality of the PROCESS_EVENT function in Sections S2 and S3.

Step 3: Check timeout and synchronization criteria The timeout criterion is met if the earliest event scheduled in Q has exceeded the *maximum simulation time*.

The synchronization criterion is met when the periods of all coupled ROs are within the specified *tolerance*. We terminate the simulation when either of the above criteria is met.

Step 4: Assign spin values At the end of the simulation, the RO phases are translated to spin values, assigning a spin of $+1$ to the reference RO. The phase difference between the RO in the A2A array and every other RO in the array is determined: if this phase difference is closer to 0 than it is to π, a spin value of $+1$ is assigned to the RO, otherwise, we assign a spin value of -1.

Algorithm 1 Simulation of an A2A array of ROs

```
Input: Timing file, circuit netlist, coupling matrix, maximum simulation time, and tolerance.
Output: A spin assignment for ROs.
// Step 1: Initialize.
initial_events ← Initialize with events on enable pins
Net2Event, PendingTrigger ← map()
for event ∈ initial_events do
    Q.add(event)
    Net2Event[event.netname] = event
end for
timeout ← False
synchronized ← False
while !timeout and !synchronized do
    // Step 2: Pop an event for processing.
    E ← Q.pop()
    PROCESS_EVENT(E, Q, Net2Event, PendingTrigger)
    // Step 3: Check timeout and synchronization criteria.
    if Q[0].arrival_time > maximum simulation time then
        timeout ← True
    end if
    if synchronization criteria met then
        // Synchronization condition defined in Section II-B of the manuscript.
        synchronized ← True
    end if
end while
// Step 4: Assign spin values
spin_vals ← Assign spins based on the phase difference of each RO with the reference RO
return spin_vals, Net2Event
```

S2. Pseudocode for the PROCESS_EVENT function

The pseudocode for the function PROCESS_EVENT is listed in Algorithm 2. The function receives an event E, the queue Q, and the maps Net2Event and PendingTrigger, and generates output event(s) which it inserts into Q. The major steps involved are as follows:

Algorithm 2 PROCESS_EVENT: Processing an event in the event queue

```
function PROCESS_EVENT(E, Q, Net2Event, PendingTrigger)
  cell = the cell instance that receives event E at its input
  remove_event = list() // List for processed events
  NE = list() // List for output events
  // Step PE1: Find the cell type of the cell that receives E as input.
  if cell is a coupling or shorting cell then
    pin = the pin of cell that event E occurs at
    // Step PE2: Find the direction of the path that E lies on.
    if pin is $h^f_{in}$ or $v^f_{in}$ then
      // Step PE3: Find an interacting event for the forward path.
      if event E' exists on other input then
        if E and E' are within a window of W then
          NE ← Calculate output events from E and E'
          remove_event.add([E, E'])
        else
          NE ← Calculate output event from E
          remove_event.add(E)
        end if
      else
        // Step PE4: Look back to find events that might result in interaction.
        net = the net connected to other input of cell
        (left, right) = (E.arrival_time - W, E.arrival_time + W)
        status = LOOK_BACK(net, (left, right), left, Net2Event)
        if status then
          PendingTrigger[net] = E
        else
          NE ← Calculate output event from E
          remove_event.add(E)
        end if
      end if
    else
      // Calculate events on the backward path.
      NE ← Calculate output event from E
      remove_event.add(E)
    end if
  else
    // enable cell
    NE ← Calculate output event from E
    remove_event.add(E)
  end if
  // Step PE5: Check if events in NE are triggers to a pending event
  if NE not empty then
    for new_event ∈ NE do
      Q.add(new_event)
      Net2Event[new_event.netname] = new_event
      if new_event.netname in PendingTrigger then
        Q.add(PendingTrigger[new_event.netname])
        PendingTrigger.pop(new_event.netname)
      end if
    end for
  end if
  // Remove consumed events
  for used_event ∈ remove_events do
    Net2Event.pop(used_event.netname)
  end for
end function
```

Step PE1: Find the cell type of the cell that receives E as input The netlist and the net name property of the event object E are used to determine the cell that receives the event at its input. If the cell is an enable cell, the output event can be calculated directly (lines 37–39), and the event is processed. Otherwise, in case of a coupling or a shorting cell, we proceed to Step PE2.

Step PE2: Find the direction of the path that E lies on If the event E occurs on a net on the reverse path (on pin h^r_{in} or v^r_{in} of a cell), there is no coupling interaction and the output events are calculated directly (lines 32–34). Otherwise, for an event on a forward path (on pin h^f_{in} or v^f_{in}), we proceed to Step PE3.

Step PE3: Find an interacting event for the forward path If the event E occurs on h^f_{in}, then only an event at v^f_{in} of the same cell can interact with E and vice-versa. The map Net2Event can be queried with the net name of the other input to look for an interacting event E'. Even if an event at the other input is not found in the map, it is possible that such an event has not yet been generated from a predecessor cell, and we proceed to Step PE4 to look for events that might still result in interaction with E. Note that the interaction window lies in a range of $\pm W$ around the arrival time of event E, and therefore this process should identify any event within this window – one that precedes or succeeds E.

If found, the switching information for events E and E' is sufficient to determine whether they interact or not, and the output events may be calculated (lines 11–18). Interacting events E and E' generate a pair of output events, while a noninteracting event generates one output event.

Step PE4: Look back to find events that might result in interaction When an event is on the other input not found in the map Net2Event, the function LOOK_BACK, described in detail in Section S3, is invoked. As described at the end of the example, for an event E at a cell C, intuitively, this procedure looks into the predecessors of C to determine whether any incoming, but as yet unprocessed, event might result in another event that interacts with E.

If such an event is found, the event E is added to the PendingTrigger map with the net name of the other input of the cell as its trigger. If not, we generate the output event from E assuming no interaction.

Step PE5: Check if output events are triggers to a pending event If E generates an event on a net that is a trigger to a pending event in the PendingTrigger map, then the pending event is ready to be processed and is added to the queue.

S3. Pseudocode for the LOOK_BACK function

The pseudocode for the function LOOK_BACK is provided in Algorithm 3. The inputs to the function are a net *event_net*, a window of arrival time (left, right), a threshold arrival time *threshold*, and the map Net2Event. LOOK_BACK is a recursive function that terminates when it encounters one of three base cases:

- The latest arrival time of an event at *event_net* is less than *threshold*. The function returns false.
- An event arrives at *event_net* within the window and can result in an interaction. The function returns true.
- An event arrives at *event_net* outside the window and will not result in an interaction. The function returns false.

Algorithm 3 LOOK_BACK: Identification of events that might interact with an event being processed

```
function LOOK_BACK(event_net, (left, right), threshold, Net2Event)
  // looks back recursively to find events that might interact
  if right < threshold then
    // Base case: looked back far enough
    return False
  end if
  if event_net is in Net2Event then
    // Base cases: Interacting or not
    if the event will arrive in (left, right) then
      return True
    else if the event will not arrive in (left, right) then
      return False
    end if
  else
    // Recursive step: need to look back further
    preceding_net = the net that can cause an event at event_net
    preceding_cell = the instance for which event_net is an output
    (nleft, nright) = (left - preceding_cell.d_max, right - preceding_cell.d_min)
    return LOOK_BACK(preceding_net, (nleft, nright), threshold, Net2Event)
  end if
end function
```

The first base case prevents us from looking at too many predecessors by comparing the latest arrival time to *threshold*. The *threshold* is assigned a value in PROCESS_EVENT which corresponds to the earliest arrival time for an interaction with the event

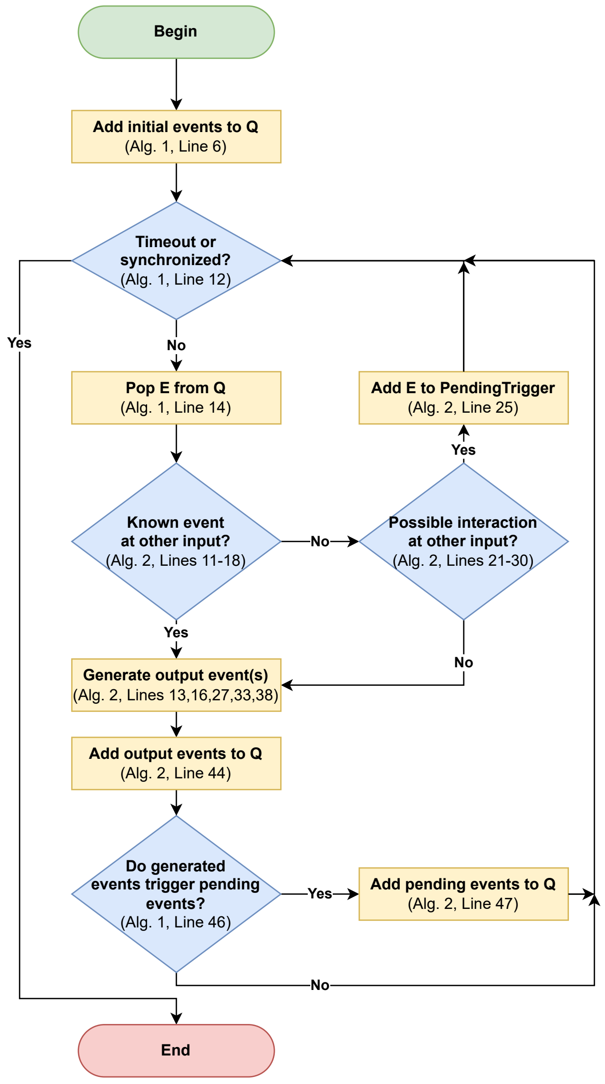

Fig. S1. A flowchart outlining the flow of control for DROID. The processes and decisions are mapped to the line numbers of the pseudocodes.

that invoked LOOK_BACK. The second and third cases require looking at the map Net2Event for an event with the key *event_net* to decide if it will arrive within the arrival time window. When none of the base cases are encountered, the function moves to the recursive step.

The predecessor of *event_net*, which we call *preceding_net* is found when an event at *event_net* does not exist in Net2Event. The cell that *preceding_net* is an input of is called *preceding_cell*. The minimum and maximum delays (d_{min} and d_{max}, respectively) for *preceding_cell* are used to calculate a window of arrival for *preceding_net*. A recursive call is made at *preceding_net* with this new window, with the recursion concluding when the interaction window has been exceeded.

S4. Flowchart and evolution of data structures

The flow of control for DROID is shown using a flowchart in Fig. S1. The first step is the initialization of events on the enable pins of the ROs that are added to Q which sorts them by their arrival times. These initial events, when processed by the simulator, cause events that are added to Q and scheduled for future processing. The events are continuously popped from Q until the array synchronizes or reaches the specified timeout. An event popped from Q can occur at the input of any of the three types of cells – *enable*, *shorting*, and *coupling cells*. The timing model of an *enable cell* is just a delay as it has no coupling circuit. The timing models for the *shorting cell* and the *coupling cell* require the simulator to detect events at the other input of the cell, as the path delays change based on the arrival times of the events.

An event at the other input of a *shorting cell* or a *coupling cell* might be readily available in Net2Event. The output event(s) can then be scheduled by determining the delays based on the interaction of events. However, if an event at the other input is not

detected in Net2Event, the simulator looks for events on other nets that might result in future interaction. In case of a possible future interaction, the processing of the popped event is postponed until another event triggers it. Otherwise, the popped event is processed assuming no interaction with other events. The generated events are added to Q and are checked if they trigger any previously postponed events, in which case the postponed events are added back to Q for processing.

The evolution of the queue Q and the maps Net2Event and PendingTrigger, as DROID processes events is shown in Fig. S2. The figure illustrates how events are generated for a few common scenarios on a small section of an A2A array assuming toy numbers. Fig. S2 (a) shows a section of an A2A array with two ROs and labeled nets that will be used for this example. For the sake of simplicity, we will concern ourselves only with the arrival times of events and not worry about their transition times or types. Net2Event tracks the nets at which an event is scheduled, with net names pointing to events. PendingTrigger maps a trigger net to a postponed event such that the postponed event is added back to Q when an event is detected at the trigger net.

The simulation begins when initial events E0 and E1 with arrivals at 10ps and 120ps, respectively, are added to Q and Net2Event, and PendingTrigger is initialized to an empty map. As E0 occurs at the input of two enable cells, its processing yields E2 and E3 at nets a_0 and w_0, respectively (Fig. S2 (b)). Output events are added to Q and Net2Event, while input events are removed from both.

The queue Q sorts events by their arrival times with the earliest occurring event at the head. As E2 and E3 arrive earlier than E1, they are placed ahead in Q (Fig. S2 (c)). E2 is popped next and is detected to occur at a shorting cell which prompts a search for an event at w_0. Net2Event contains E3 at w_0 so the arrival times of E2 and E3 are compared to check if they interact. As mentioned in the manuscript, two events interact when their arrival times lie within the interaction window W.

An estimate for W can be obtained as follows. Let τ_c be the largest transition time (rise or fall) at a coupled net and C_{max} be the largest coupling coefficient implemented by the coupling cell. Then, C_{max} successive stages with transitions no larger than τ_c bring an estimate of W to be $\tau_c \times C_{max}$. In this example, E2 and E3 arrive at the same time and are therefore, interacting events that are both consumed to generate E4 and E5 at nets b_0 and x_0, respectively. In addition to its removal from Net2Event, E3 must be removed from Q despite not having been popped.

E4 is the next event to be popped from Q (Fig. S2 (d)) and requires an event at w_1 to determine interaction. As Net2Event has no event mapped to w_1, LOOK_BACK is invoked. Event E1 (yet to be processed) is found capable of generating an event at w_1 in the future that might interact with E4. Thus, E4 is added to PendingTrigger until an event occurs on w_1 that would trigger its readdition to Q. Similarly, E5 is also added to PendingTrigger until an event occurs on a_1 (Fig. S2 (e)). Events added to PendingTrigger are removed from Q but not from Net2Event.

The next event to be popped from Q is E1. Like E0, E1 occurs at the input of two enable cells and generates E6 and E7 at nets a_1 and w_1, respectively. Recall that the processing of E4 and E5 was postponed until events on a_1 and w_1 are known. As E6 and E7 are scheduled, they also trigger the addition of E4 and E5 back to Q (Fig. S2 (f)). E4 is popped from Q again, and now E7 is available in Net2Event. For this example, we assume that the E4 and E7 are deemed non-interacting based on their arrival times, and only E4 is consumed to generate E8 on net c_0. E7 stays in Q and Net2Event (Fig. S2 (g)).

The processing of E5 is similar to that of E4. E5 prompts a search for an event on a_1. Event E6 is detected in Net2Event and we assume the arrival times of E5 and E6 make them non-interacting events. E5 is consumed alone to generate E9 on y_0 and E6 awaits its turn (Fig. S2 (h)).

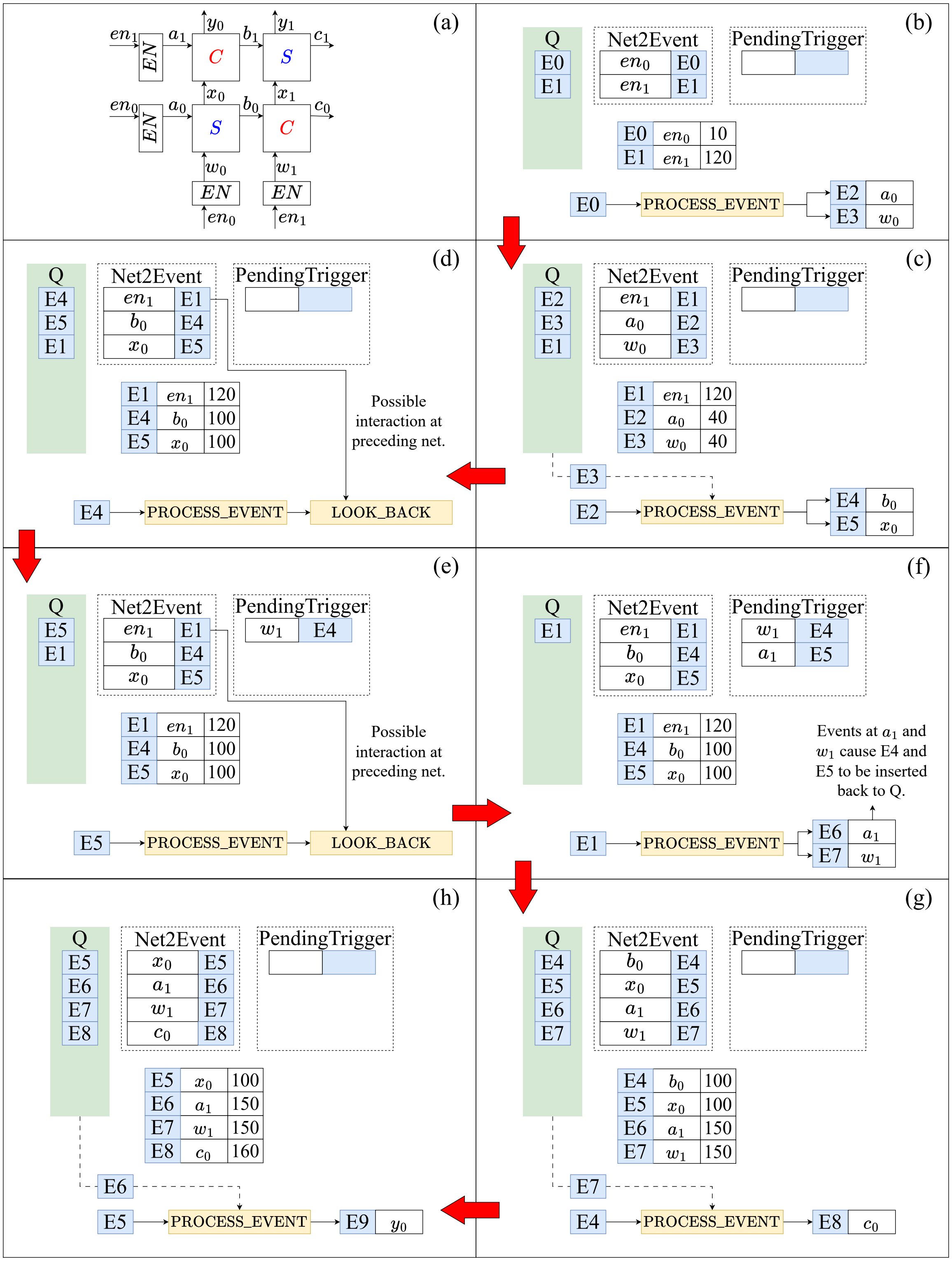

Fig. S2. (a) A section of the A2A array with cell types and net names. (b) Populating Q with initial events E0 and E1. E0 is popped and is processed to yield E2 and E3. (c) Interacting events E2 and E3 are consumed to produce E4 and E5. (d) E4 is popped for processing but Net2Event has no event at w_1. E1 might cause an interacting event in the future so processing of E4 is stalled by adding it to PendingTrigger until an event occurs on w_1. (e) Like E4, E5 is added to PendingTrigger until an event occurs on a_1. (f) E1 is popped and processed. It generates E6 and E7 on nets a_1 and w_1, respectively. These events trigger the pending events E4 and E5, causing them to be added back to Q. (g) E4 is popped again, but this time with E7 in Net2Event. Due to the events not interacting with each other, E4 is consumed to generate a single event E8 while E7 awaits its turn. (h) E5 can be processed as E6 is in Net2Event. E5 is consumed to generate E9 and E6 awaits its turn.

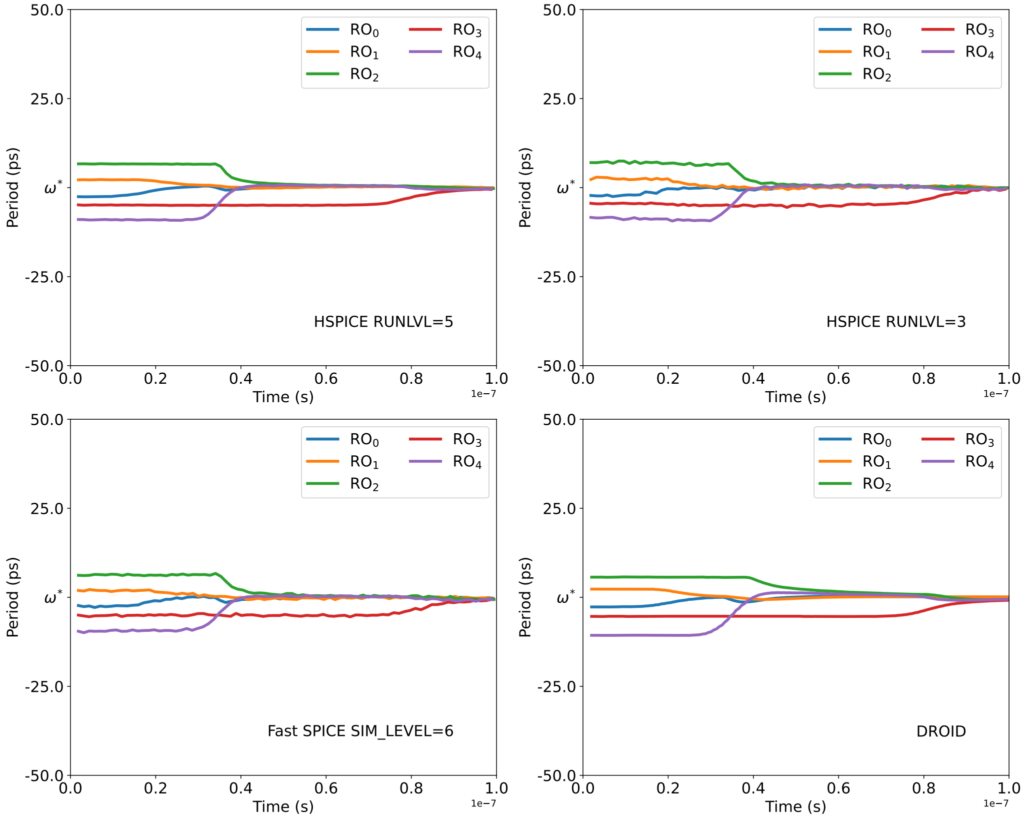

Fig. S3. Period waveforms from HSPICE, fast SPICE, and DROID for a 5×5 A2A array. HSPICE RUNLVL=5 (top left) is the most accurate but is also the slowest. HSPICE RUNLVL=3 (top right) achieves a reasonable level of accuracy at almost half the runtime. Fast SPICE at SIM_LEVEL=6 (bottom left) resembles HSPICE RUNLVL=3 and is about an order of magnitude faster. DROID (bottom right) is the fastest and resembles HSPICE.

S5. Accuracy and Runtime Comparisons with SPICE Tools

We use an accurate HSPICE simulation of a 5×5 A2A as a reference to compare the accuracy of DROID and fast SPICE in Fig. S3. Fig. S3 illustrates the change in the periods of the five ROs over time as predicted by various simulators. The waveforms obtained from HSPICE at RUNLVL=5 (top left in Fig. S3) correspond to an accurate simulation. Reducing HSPICE accuracy to RUNLVL=3 (top right in Fig. S3) halves the runtime while still achieving acceptable accuracy. Fast SPICE simulation at SIM_LEVEL=6 (bottom left in Fig. S3) achieves similar results as HSPICE RUNLVL=3 but is nearly an order of magnitude faster. Lowering the SIM_LEVEL in fast SPICE any further was observed to reduce the accuracy to lower-than-acceptable levels. DROID (bottom right in Fig. S3) predicts a similar evolution of the array at a fraction of the runtime.

Table S1 summarizes the simulation runtimes for array sizes of 5×5, 20×20, and 50×50, with DROID speedups in parentheses. The arrays were simulated for 100ns using DROID, fast SPICE, and HSPICE. DROID is always run on a single core. HSPICE was run with eight cores but at different accuracy levels. An accurate simulation of a 50×50 array for 100ns in HSPICE (corresponding to RUNLVL=5) takes more than 16 hours. Reducing the accuracy (corresponding to RUNLVL=3) still requires more than 3 hours. Both simulations would take longer if run on a single core. Fast SPICE is significantly faster than HSPICE at the cost of accuracy. Simulating a 50×50 array for 100ns in fast SPICE at the highest accuracy (SIM_LEVEL=6) with one core required about 40 minutes. Increasing the number of cores to eight improved the runtime to 14 minutes but it was not an eight-fold improvement. As can be seen, DROID is two orders of magnitude faster than fast SPICE on a realistic A2A array.

TABLE S1
Simulation runtimes for various array sizes using DROID, fast SPICE, and HSPICE.

**Array size**	5×5	20×20	50×50
**# MOSFETs**	3100	52000	328000
**DROID** (1 core)	2.1s	3.5s	7.9s
**Fast SPICE** (SIM_LEVEL=6, 1 core)	34s (**16**×)	283s (**81**×)	0.68h (**310**×)
**Fast SPICE** (SIM_LEVEL=6, 8 cores)	24s (**11**×)	111s (**32**×)	0.24h (**109**×)
**HSPICE** (RUNLVL=3, 8 cores)	142s (**68**×)	0.52h (**534**×)	3.40h (**1549**×)
**HSPICE** (RUNLVL=5, 8 cores)	262s (**125**×)	1.04h (**1072**×)	16.33h (**7441**×)
